# Broadband Near-Infrared Luminescence in Lead Germanate Glass Triply Doped with Yb^3+^/Er^3+^/Tm^3+^

**DOI:** 10.3390/ma14112901

**Published:** 2021-05-28

**Authors:** Wojciech A. Pisarski, Joanna Pisarska, Radosław Lisiecki, Witold Ryba-Romanowski

**Affiliations:** 1 Institute of Chemistry, University of Silesia, Szkolna 9 Street, 40-007 Katowice, Poland; joanna.pisarska@us.edu.pl; 2 Institute of Low Temperature and Structure Research, Polish Academy of Sciences, Okólna 2 Street, 50-422 Wrocław, Poland; r.lisiecki@int.pan.wroc.pl (R.L.); w.ryba-romanowski@int.pan.wroc.pl (W.R.-R.)

**Keywords:** lead germanate glasses, rare earth ions, near-infrared luminescence

## Abstract

This paper deals with broadband near-infrared luminescence properties of lead germanate glass triply doped with Yb^3+^/Er^3+^/Tm^3+^. Samples were excited at 800 nm and 975 nm. Their emission intensities and lifetimes depend significantly on Er^3+^ and Tm^3+^ concentrations. For samples excited at 800 nm, broadband emissions corresponding to the overlapped ^3^H_4_ → ^3^F_4_ (Tm^3+^) and ^4^I_13/2_ → ^4^I_15/2_ (Er^3+^) transitions centered at 1.45 µm and 1.5 µm was identified. Measurements of decay curves confirm reduction of ^3^H_4_ (Tm^3+^), ^2^F_5/2_ (Yb^3+^) and ^4^I_13/2_ (Er^3+^) luminescence lifetimes and the presence of energy-transfer processes. The maximal spectral bandwidth equal to 269 nm for the ^3^F_4_ → ^3^H_6_ transition of Tm^3+^ suggests that our glass co-doped with Yb^3+^/Er^3+^/Tm^3+^ is a good candidate for broadband near-infrared emission. The energy transfer from ^4^I_13/2_ (Er^3+^) to ^3^F_4_ (Tm^3+^) and cross-relaxation processes are responsible for the enhancement of broadband luminescence near 1.8 µm attributed to the ^3^F_4_ → ^3^H_6_ transition of thulium ions in lead germanate glass under excitation of Yb^3+^ ions at 975 nm.

## 1. Introduction

Lanthanide triply doped inorganic glass is an excellent candidate for ultra-wide near-infrared (NIR) luminescence covering the 1200–2100 nm spectral range [1]. Systematic studies demonstrate that bands of selected lanthanide ions located in the NIR region are quite well overlapped, making an important contribution to broadband luminescence. Several different glasses triply doped with lanthanide ions were proposed as efficient systems emitting NIR radiation. There are glass systems containing Nd^3+^/Er^3+^/Tm^3+^ [2], Nd^3+^/Er^3+^/Pr^3+^ [3], Yb^3+^/Er^3+^/Pr^3+^ [4], and Yb^3+^/Ce^3+^/Er^3+^ [5], important for the optical telecommunication window (1200–1650 nm) as well as Yb^3+^/Tm^3+^/Ho^3+^ [6] and Yb^3+^/Er^3+^/Ho^3+^ [7] for NIR laser sources at about 2 µm. From the experimental tests of different glass systems, it can be concluded that low-phonon inorganic glasses triply doped with lanthanide ions are promising for numerous applications in the field of infrared photonics and laser technology such as optical telecommunications, broadband near-infrared fiber amplifiers, solid-state laser sources, and other optoelectronic devices.

Among fully amorphous systems and glass–ceramic materials, the glasses with suitable Yb^3+^/Er^3+^/Tm^3+^ ion combination are interesting mainly for two purposes. Three simultaneously observed emission bands assigned to the ^1^G_4_ → ^3^H_6_ transition of Tm^3+^ (blue band), the ^2^H_11/2_,^4^S_3/2_ → ^4^I_15/2_ (green band), and ^4^F_9/2_ → ^4^I_15/2_ (red band) transitions of Er^3+^ under direct excitation of Yb^3+^ at 975 nm favor the generation of white light through a well-known mechanism of up-conversion process. To obtain white emission, the concentrations of lanthanide ions (Yb^3+^, Er^3+^, Tm^3+^) and their relative molar ratios should be optimized, and the pump power of the up-conversion process should also not be ignored. Thus, white up-conversion luminescence of Yb^3+^/Er^3+^/Tm^3+^ ions in glass [8,9,10] and glass–ceramic materials containing fluoride nanocrystals YF_3_ [11,12] were successfully observed. These effects were also examined for glass with silver nanoparticles [13,14].

Alternatively to the white up-conversion process, glass with Yb^3+^/Er^3+^/Tm^3+^ ions is also attractive for near-infrared radiation. Three near-infrared emission bands related to the ^4^I_13/2_ → ^4^I_15/2_ transition of Er^3+^ at 1.5 µm, the ^3^H_4_ → ^3^F_4_ (1.45 µm), and ^3^F_4_ → ^3^H_6_ (1.8 µm) transitions of Tm^3+^ can be observed under excitation at 800 nm or 975 nm. However, these phenomena have rarely been examined. For systems with Yb^3+^/Er^3+^/Tm^3+^, near-infrared luminescence properties were limited to multicomponent TeO_2_–ZnO–WO_3_–TiO_2_–Na_2_O glass [15] and oxyfluoride silicate glass ceramics containing nanocrystals PbF_2_ [16,17].

This paper concerns broadband near-infrared luminescence in lead germanate glass triply doped with Yb^3+^/Er^3+^/Tm^3+^. To the best of our knowledge, these aspects have not been studied before. In general, lead germanate-based glass doped with lanthanide ions and its structure and optical properties are well documented in the literature [18,19,20,21,22]. They are an alternative candidate to tellurite glass for nonlinear fiber applications [23]. In recent years, luminescence properties of lead germanate glass singly doped with Yb^3+^ [24,25,26], Er^3+^ [27,28,29], and Tm^3+^ [30,31] have been well presented and discussed. Special attention has been paid to lead germanate glass co-doped with Yb^3+^/Er^3+^ [32,33,34] and Yb^3+^/Tm^3+^ [35,36], and their up-conversion luminescence processes. Further experimental studies revealed that lead germanate glass triply doped with Yb^3+^/Tm^3+^/Ho^3+^ [37,38,39], Yb^3+^/Tm^3+^/Nd^3+^ [40], and Yb^3+^/Tm^3+^/Er^3+^ [41] ions are promising materials for up-conversion luminescence applications.

## 2. Materials and Methods

Lead germanate glass triply doped with rare earths with chemical formula [mol%] 45PbO-45GeO_2_-(5-x-y)Ga_2_O_3_-5Yb_2_O_3_-xTm_2_O_3_-yEr_2_O_3_ (x and y = 0, 0.5, 1.5) were prepared. Glass codes are as follows: 0.5 Tm-0.5 Er; 0.5 Tm-1.5 Er; 1.5Tm-0.5Er; and 1.5Tm-1.5Er. They were compared to glass samples co-doped with Yb^3+^/Tm^3+^ referred to as 0.5 Tm and 1.5 Tm, respectively. Precursor metal oxides of high purity (99.99%) were mixed in an agate ball mill. The batch of the starting reagents was placed into a ceramic crucible and the melt was directly poured onto a preheated steel plate. Melting temperature and time are as follows: T = 1100 °C, t = 0.5 h. To reduce the internal stresses, the obtained glass was annealed below the glass transition temperature. For the optical measurements, the glass samples were adequately cut and polished to achieve excellent transparency. Eventually, glass samples with dimensions of 10 × 10 mm and thickness of 2 mm were obtained. Luminescence spectra measurements were carried out using a laser system, which consists of an optical parametric oscillator coupled with Nd:YAG (Continuum Surelite OPO and SLI-10 Nd:YAG laser, Santa Clara, CA, USA), 1 m double grating monochromator, a photomultiplier, boxcar integrator (Stanford SRS250), and oscilloscope (Tektronix model TDS3052, two-channel color digital phosphor oscilloscope, 500 MHz, Tektronix Inc., Beaverton, OR, USA). The investigated glass was mounted in a sample holder and an excitation beam was directed on the sample side edge from a distance of 10 cm. To avoid the signal saturation, the excitation beam was directed perpendicular to a monochromator aperture and the laser spot on the sample was no higher than 2 mm. The resulting signal was collected from the greatest volume of the glass samples using a convex 75 mm lens. The excitation laser power both for 800 nm and 975 nm was set at 450 mW. Resolution for luminescence spectra measurements was ±0.2 nm. Decays were registered with an accuracy of ±2 µs. For the luminescence decay curve measurements, the excitation pulse laser duration was 4 ns, and the pulse energies depending on the applied wavelengths were between 20–40 mJ. To record the NIR transients the InGaAs Hamamatsu and a cooled InSb Janson J10D detectors were used. Moreover, Schott optical long-pass filters RG780, RG850, and RG1000 were employed. The experimental lifetimes of the ^3^F_4_ (Tm^3+^), ^3^H_4_ (Tm^3+^), ^4^I_13/2_ (Er^3+^), and ^2^F_5/2_ (Yb^3+^) luminescent levels have been measured at the following adequate wavelengths: 1780 nm, 815 nm, 1530 nm, and 982 nm.

## 3. Results and Discussion

Luminescence properties of lead germanate glass triply doped with Yb^3+^/Er^3+^/Tm^3+^ were examined in two NIR ranges, where emission bands of Tm^3+^ and/or Er^3+^ occur. The first spectral region (1200–1675 nm) is associated with the so-called telecommunication window. Several inorganic glasses were tested to achieve optical amplification covering the S-band (1460–1530 nm), C-band (1530–1565 nm), L-band (1565–1625 nm), and U-band (1625–1675 nm). In this near-infrared range, the spectrum consists of luminescence bands due to characteristic ^3^H_4_ → ^3^F_4_ (Tm^3+^) and ^4^I_13/2_ → ^4^I_15/2_ (Er^3+^) electronic transitions, which are relevant for the design of S-band and C+L-band amplifiers [42]. The second spectral region discussed here deals with a broadband NIR emission at 1800 nm corresponding to ^3^F_4_ → ^3^H_6_ transition of Tm^3+^.

**Figure 1:**
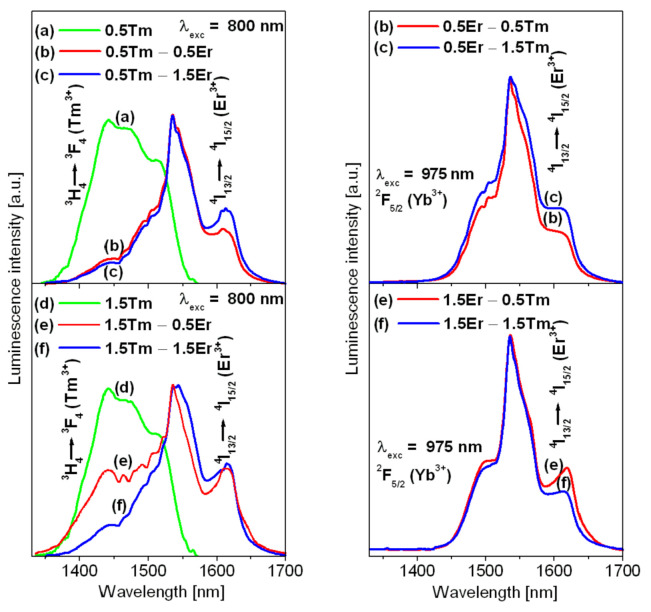
Normalized NIR luminescence spectra for lead germanate glass containing Yb^3+^/Er^3+^/Tm^3+^ and Yb^3+^/Tm^3+^ ions excited at 800 nm (**left**) and 975 nm (**right**).

Figure 1 presents luminescence spectra for lead germanate glass triply doped with Yb^3+^/Er^3+^/Tm^3+^. The spectra were compared to those for samples co-doped with Yb^3+^/Tm^3+^. To understand the energy-transfer processes, their mechanisms, and the interactions between lanthanide ions, the lead germanate glass with various Tm^3+^ and Er^3+^ concentrations was excited at 800 nm and 975 nm.

Spectra measured in the 1330–1700 nm range were normalized to compare their emission profiles and bandwidth referred to as full width at half maximum (FWHM). For glass samples excited at 800 nm, the spectra showed emission bands centered at about 1450 nm and 1530 nm, which are assigned to the ^3^H_4_ → ^3^F_4_ (Tm^3+^) and ^4^I_13/2_ → ^4^I_15/2_ (Er^3+^) transitions of lanthanides. Owing to some excellent papers published previously [43,44,45,46], the shoulder near 1650 nm in sample 1.5 Tm–0.5 Er belongs to the short-wavelength tail of the emission due to the ^3^F_4_ → ^3^H_6_ transition of Tm^3+^. The relative integrated intensities of NIR emission bands depend on Er^3+^ and Tm^3+^ concentrations. In particular, the changes in emission profiles and bandwidths are clearly visible for glass samples with higher Tm^3+^ (1.5 mol%) concentration. In contrast to glass samples with low (0.5 mol%) concentration, the intensity of the NIR emission band due to the ^3^H_4_ → ^3^F_4_ transition of Tm^3+^ decreases with increasing Er^3+^ concentration. The emission bandwidth for glass samples assigned as 1.5 Tm–0.5 Er is close to 130 nm. It is in a good agreement with the value of FWHM equal to 138 nm, which was obtained for similar germanate glass co-doped with Er^3+^/Tm^3+^ [47]. For glass sample 1.5 Tm–0.5 Er, emissions of Tm^3+^ and Er^3+^ ions are quite well overlapped giving contribution to broadband near-infrared radiation related to the S+C+L-bands of the optical telecommunication. These effects are not observed when glass samples triply doped with Yb^3+^/Er^3+^/Tm^3+^ ions were excited at 975 nm. In this case, the emission band with its typical profile for the ^4^I_13/2_ → ^4^I_15/2_ transition of Er^3+^ ions was measured under direct excitation of Yb^3+^ ions. The values of FWHM are about 55 nm and depend slightly on Tm^3+^ and Er^3+^ concentrations.

**Figure 2:**
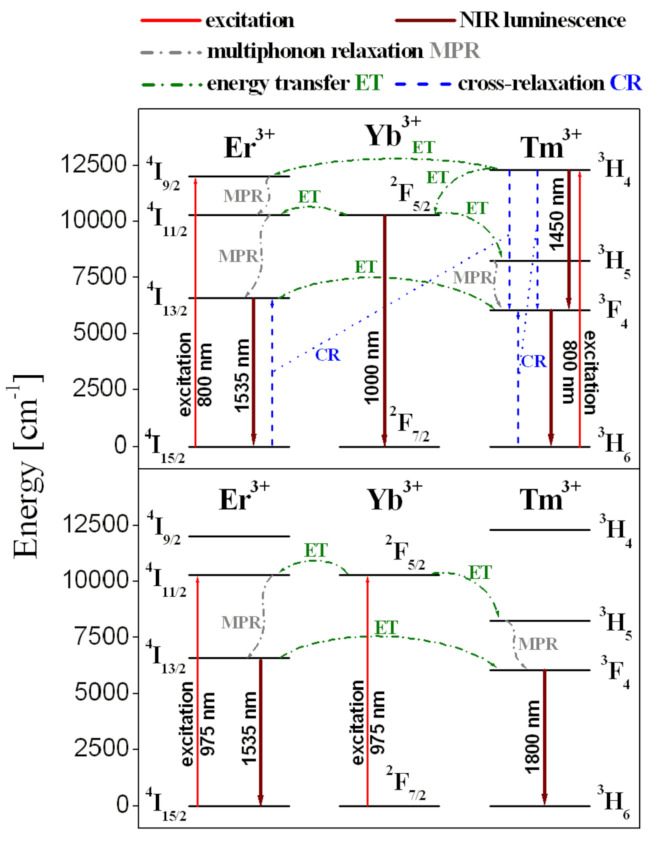
Energy level diagrams for lead germanate glass triply doped with Yb^3+^/Er^3+^/Tm^3+^ ions excited at 800 nm (**top**) and 975 nm (**bottom**). All transitions and processes are also indicated.

According to the partial energy level diagram presented in Figure 2, several energy-transfer mechanisms for the studied glass excited at 800 nm and 975 nm are proposed. When a glass sample is excited directly at 800 nm, both the ^3^H_4_ (Tm^3+^) and ^4^I_9/2_ (Er^3+^) states are simultaneously populated from their ground states. Part of the excitation energy relaxes radiatively from the ^3^H_4_ state and contributes greatly to near-infrared emissions at about 1.45 µm and 1.8 µm, which are associated with ^3^H_4_ → ^3^F_4_ and ^3^F_4_ → ^3^H_6_ transitions of Tm^3+^. At the same time, the ^3^H_4_ state is quite efficiently depopulated by the nearly resonant energy-transfer process to the ^4^I_9/2_ state of Er^3+^ and non-resonant energy-transfer process to the ^2^F_5/2_ state of Yb^3+^. Thus, near-infrared luminescence at about 1 µm due to the ^2^F_5/2_ → ^2^F_7/2_ transition of Yb^3+^ can be observed (not presented here). Additionally, energy is transferred non-radiatively from the ^2^F_5/2_ (Yb^3+^) to the ^4^I_11/2_ (Er^3+^) and ^3^H_5_ (Tm^3+^) states of lanthanides. The presence of both phonon-assisted energy-transfer processes ^3^H_4_ (Tm^3+^) → ^2^F_5/2_ (Yb^3+^) and ^2^F_5/2_ (Yb^3+^) → ^3^H_4_ (Tm^3+^) was confirmed in Yb^3+^/Tm^3+^ co-doped tellurite glasses [48].

During direct excitation of glass sample at 800 nm, depopulation of the ^4^I_9/2_ (Er^3+^) state is very fast by multiphonon relaxation via ^4^I_11/2_ state to the ^4^I_13/2_ state, from which near-infrared emission at 1.5 µm assigned to ^4^I_13/2_ → ^4^I_15/2_ transition of Er^3+^ occurs. The first excited ^4^I_13/2_ state of erbium is also depopulated non-radiatively and part of the excitation energy is transferred to thulium due to the following nearly resonant energy-transfer process ^4^I_13/2_ (Er^3+^) → ^3^F_4_ (Tm^3+^). Consequently, the enhanced NIR emission at 1.8 µm due to the ^3^F_4_ → ^3^H_6_ transition of Tm^3+^ can be observed. However, the most important non-radiative transitions that contribute to quenching (at 1.45 µm) and enhancing (near 1.8 µm) the near-infrared luminescence of Tm^3+^ are related to two cross-relaxation processes [45]: [^3^H_4_ (Tm^3+^) + ^3^H_6_ (Tm^3+^)] → [(^3^F_4_ (Tm^3+^) + ^3^F_4_ (Tm^3+^)] and [^3^H_4_ (Tm^3+^) + ^4^I_15/2_ (Er^3+^)] → [^3^F_4_ (Tm^3+^) + ^4^I_13/2_ (Er^3+^)].

When a glass sample is excited at 975 nm the ^2^F_5/2_ state of Yb^3+^ ions is quite well populated and then the excitation energy relaxes non-radiatively to the ^4^I_11/2_ (Er^3+^) and ^3^H_5_ (Tm^3+^) states by nearly resonant and non-resonant (phonon-assisted) energy-transfer process, respectively. In the next step, multiphonon relaxation contributes to the efficient population of lower-energy ^4^I_13/2_ (Er^3+^) and ^3^F_4_ (Tm^3+^) states. Consequently, near-infrared emission bands at 1.5 µm and 1.8 µm corresponding to the ^4^I_13/2_ → ^4^I_15/2_ (Er^3+^) and ^3^F_4_ → ^3^H_6_ (Tm^3+^) transitions of lanthanides are observed under excitation of Yb^3+^ ions at 975 nm.

**Figure 3:**
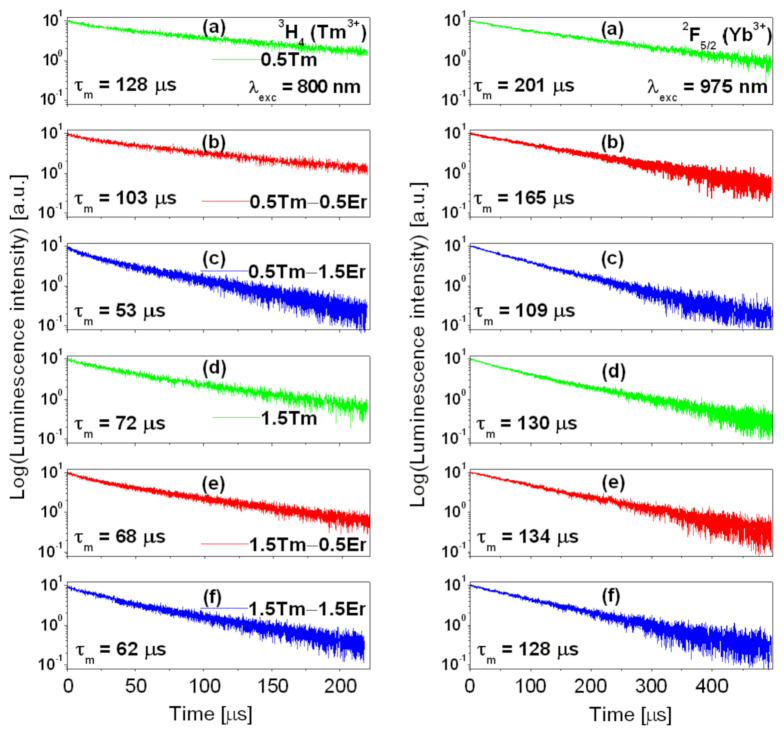
Luminescence decays from the ^3^H_4_ (Tm^3+^) and ^2^F_5/2_ (Yb^3+^) excited states.

Figure 3 shows luminescence decays from the ^3^H_4_ (Tm^3+^) and ^2^F_5/2_ (Yb^3+^) states, which were measured for glass samples excited at 800 nm and 975 nm, respectively. All decay curves exhibit a slight deviation from the single-exponential function.

For luminescence decays measured under 800 nm excitation, the curves for both simultaneously and resonantly excited states ^3^H_4_ (Tm^3+^) and ^4^I_9/2_ (Er^3+^) should be observed, because the positions of these states on the energy level diagram are nearly the same. However, it is experimentally proved that the ^4^F_9/2_ lifetime of Er^3+^ ions is one or two magnitudes of order lower than the ^3^H_4_ lifetime of Tm^3+^ ions due to the very fast non-radiative process to the lower-lying ^4^I_11/2_ (Er^3+^) state by the efficient multiphonon relaxation, and as a result its contribution to the overall luminescence decay is negligible [49]. Thus, decays measured from the ^3^H_4_ (Tm^3+^) state should be reduced. Luminescence lifetimes calculated based on decay curves should be shortened due to the depopulation of ^3^H_4_, the state of Tm^3+^ ion, and the presence of the energy-transfer process ^3^H_4_ (Tm^3+^) → ^4^I_9/2_ (Er^3+^). Luminescence decay analysis confirms this hypothesis. The results are shown in Table 1.

For glass samples with low Tm^3+^ concentration (0.5 mol%), the measured ^3^H_4_ lifetime decreased from 128 µs (0.5 Tm) to 103 µs (0.5 Tm − 0.5 Er) and 53 µs (0.5 Tm − 1.5 Er) in the presence of Er^3+^ ions, suggesting an efficient energy-transfer process from a ^3^H_4_ (Tm^3+^) state to a ^4^I_9/2_ (Er^3+^) state. The reduction of luminescence lifetime is considerably lower for glass samples with relatively higher Tm^3+^ concentration (1.5 mol%). Similar effects were also obtained for decays from the ^2^F_5/2_ excited state of Yb^3+^. The measured ^2^F_5/2_ luminescence lifetime of Yb^3+^ is reduced from 201 µs (0.5 Tm) to 165 µs (0.5 Tm − 0.5 Er) and 109 µs (0.5 Tm − 1.5 Er) in the presence of Er^3+^ ions, whereas its value 131 ± 3 µs is nearly unchanged for glass samples with a higher Tm^3+^ concentration. The same situation was observed during measurements of luminescence decays from the ^4^I_13/2_ excited state of Er^3+^ ions. For lead germanate glass triply doped with Yb^3+^/Er^3+^/Tm^3+^ ions, the ^4^I_13/2_ decay is shortened with increasing Er^3+^ concentration, but changes in luminescence lifetimes are greater for glass samples containing lower (0.5 mol%) than higher (1.5 mol%) Tm^3+^ concentration (see Table 1). This indicates that processes of energy migration between the same lanthanide ions Ln^3+^-Ln^3+^ dominate the energy-transfer processes from Tm^3+^ to Er^3+^ or Yb^3+^ to Er^3+^/Tm^3+^ ions when activator concentrations are high.

**Figure 4:**
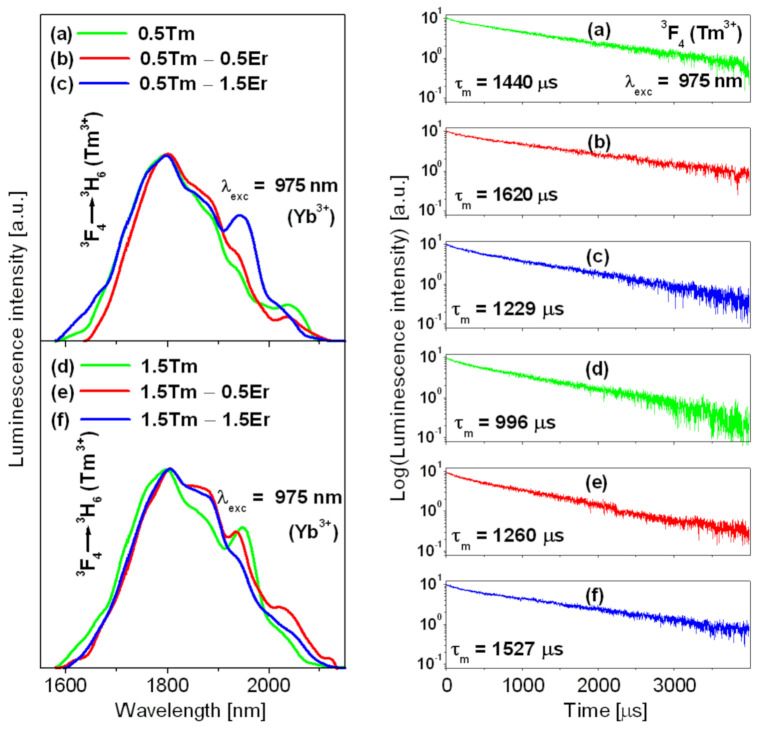
Normalized NIR luminescence spectra (**left**) and their decays (**right**) measured for lead germanate glass containing Yb^3+^/Er^3+^/Tm^3+^ and Yb^3+^/Tm^3+^ ions excited at 975 nm.

Finally, NIR luminescence spectra were measured for lead germanate glass triply doped with Yb^3+^/Er^3+^/Tm^3+^ and then compared to glass samples co-doped with Yb^3+^/Tm^3+^. The results are presented in Figure 4.

To compare the emission bandwidth, the spectra measured under 975 nm excitation were normalized. The observed near-infrared luminescence band centered at about 1.8 µm corresponds to the ^3^F_4_ → ^3^H_6_ transition of Tm^3+^. Luminescence decays from the upper ^3^F_4_ state of Tm^3+^ ions were also registered. Interestingly, the values of FWHM for samples with low Tm^3+^ content are close to 204 nm (0.5 Tm), 206 nm (0.5 Tm − 0.5 Er), and 269 nm (0.5 Tm − 1.5 Er). The later value for 0.5 Tm − 1.5 Er sample is consistent with previous results (FWHM = 270 nm for band at 1.8 µm) obtained for calcium boroaluminate glass co-doped with Er^3+^/Tm^3+^ [50]. It suggests that our glass system with Yb^3+^/Er^3+^/Tm^3+^ ions is a quite good candidate for broadband emission at 1.8 µm. Further spectroscopic analysis indicates that the emission bandwidth is reduced from 267 nm (1.5 Tm) to 241 nm (1.5 Tm − 0.5 Er) and 233 nm (1.5 Tm − 1.5 Er) in the presence of Er^3+^ ions in glass samples containing higher Tm^3+^ concentration.

The previously published results for Yb^3+^/Tm^3+^ co-doped glass pointed out that the co-doping concentrations of Tm^3+^ and Yb^3+^ should be relatively high to obtain an efficient near-infrared luminescence at 1.8 µm [8]. When the concentration of Yb^3^^+^ is relatively high (5 mol%) and constant in our all glass samples triply doped with Yb^3+^/Er^3+^/Tm^3+^ ions, the excitation energy transfer is favored by processes of energy migration Yb^3^^+^–Yb^3+^ (^2^F_5*/*2_,^2^F_7*/*2_ → ^2^F_7*/*2_,^2^F_5*/*2_), Er^3^^+^–Er^3^^+^ (^4^I_15/2_,^4^I_13/2_ → ^4^I_13/2_,^4^I_15/2_) and Tm^3^^+^–Tm^3^^+^ (^3^H_6_,^3^F_4_ → ^3^F_4_,^3^H_6_) with increasing (Er^3+^ and Tm^3+^) activators concentrations. Our experimental observations from luminescence spectra and their decays confirm that the energy-transfer processes depend significantly on both Er^3+^ and Tm^3+^ concentrations. Luminescence decay analysis for samples containing low Tm^3+^ concentration indicates that the ^3^F_4_ lifetime increases from 1440 µs (0.5 Tm) to 1620 µs (0.5 Tm − 0.5 Er) in the presence of Er^3+^ suggesting the energy transfer from erbium to thulium ions and the enhancement of near-infrared emission at 1.8 µm. Then, the measured ^3^F_4_ lifetime decreases to 1229 µs (0.5 Tm − 1.5 Er) with further increasing Er^3+^ concentration. This behavior is related to the increasing role of energy migration Er^3^^+^–Er^3^^+^ (^4^I_15/2_,^4^I_13/2_ → ^4^I_13/2_,^4^I_15/2_). The enhancement of ^3^F_4_ lifetime in the presence of Er^3+^ is also observed for glass samples containing higher Tm^3+^ content, but this trend is completely different. The measured ^3^F_4_ luminescence lifetime increases from 996 µs (1.5 Tm) to 1260 µs (1.5 Tm − 0.5 Er) and 1527 µs (1.5 Tm − 1.5 Er) in the presence of Er^3+^. It corroborates the results obtained from luminescence spectra. In fact, the intensity of the emission band at 1.8 µm grows with increasing Er^3+^ concentration. In this case, the cross-relaxation processes [^3^H_4_ (Tm^3+^) + ^3^H_6_ (Tm^3+^)] → [(^3^F_4_ (Tm^3+^) + ^3^F_4_ (Tm^3+^)], and [^3^H_4_ (Tm^3+^) + ^4^I_15/2_ (Er^3+^)] → [^3^F_4_ (Tm^3+^) + ^4^I_13/2_ (Er^3+^)] are enhanced by increasing Tm^3+^ concentration providing an important contribution to the efficient population of the upper ^3^F_4_ excited state and then the improved near-infrared luminescence at 1.8 µm due to the ^3^F_4_ → ^3^H_6_ transition of Tm^3+^ ions. At this moment, it should also be mentioned that the up-conversion luminescence mechanisms including the ground state absorption (GSA) and the excited state absorption (ESA) processes play a significant role in the excited state relaxation between lanthanides in lead germanate glass and should not be ignored. The intensities of NIR emission bands around 1.5 µm (Er^3+^) and 1.8 µm (Tm^3+^) can be diminished by losses of the excited state absorption process (ESA) due to the ^4^I_13/2_ → ^4^F_9/2_ transition of Er^3+^ ions. It suggests that thulium ions favor the energy-transfer processes between ^4^I_13/2_ (Er^3+^) and ^3^F_4_ (Tm^3+^) states by decreasing the mechanism of the ESA process due to the ^4^I_13/2_ → ^4^F_9/2_ transition of Er^3+^ and consequently the improvement of near-infrared emission at 1.8 µm, independently on single- or dual-wavelength pumping schemes [51]. However, these phenomena will be examined in a separate work.

## 4. Conclusions

Lead germanate glass triply doped with Yb^3+^/Er^3+^/Tm^3+^ has been examined for near-infrared emission applications. Glass samples were excited at 800 nm and 975 nm. Their emission intensities and lifetimes depend critically on activator (Er^3+^ and Tm^3+^) concentrations. Broadband emission with its spectral bandwidth FWHM equal to 130 nm covering the S+C+L-bands corresponding to the ^3^H_4_ → ^3^F_4_ (Tm^3+^) and ^4^I_13/2_ → ^4^I_15/2_ (Er^3+^) transitions was measured for glass samples containing 1.5 mol% Tm^3+^ and 0.5 mol% Er^3+^ under 800 nm excitation. The energy transfer from the ^4^I_13/2_ (Er^3+^) state to the ^3^F_4_ (Tm^3+^) state and cross-relaxation processes make an important contribution to broadband emissions near 1.8 µm assigned to the ^3^F_4_ → ^3^H_6_ transition of Tm^3+^. The highest emission bandwidth for a glass sample containing 0.5 mol% Tm^3+^ and 1.5 mol% Er^3+^ is close to 269 nm. Based on luminescence decay measurements, the energy-transfer processes, and their mechanisms between the excited states of lanthanide ions in lead germanate glass were confirmed.

Our studies indicate that luminescence decays from the ^3^H_4_ (Tm^3+^) and ^2^F_5/2_ (Yb^3+^) excited states measured for lead germanate glass in the presence of Er^3+^ were shortened compared to Yb^3+^/Tm^3+^ co-doped glass samples. The changes in luminescence lifetimes are greater for glass samples containing low (0.5 mol%) than higher (1.5 mol%) Tm^3+^ concentration. The same effects have been observed for the ^4^I_13/2_ lifetimes of Er^3+^. Further investigations revealed that the luminescence lifetimes related to the ^3^F_4_ → ^3^H_6_ transition of Tm^3+^ are enhanced for glass samples with the presence of Er^3+^ ions. It suggests that Yb^3+^/Er^3+^/Tm^3+^ triply doped lead germanate glass is a promising host material for broadband near-infrared luminescence at 1.8 µm. This was discussed based on the energy level diagram including all transitions and processes present in lead germanate glass with Yb^3+^/Er^3+^/Tm^3+^.

## Figures and Tables

**Table 1:** Luminescence lifetimes for the excited states of lanthanide ions in lead germanate glass calculated based on decay curve measurements.

Glass Code	Luminescence Lifetime (µs)			
	^3^H_4_ (Tm^3+^)	^2^F_5/2_ (Yb^3+^)	^4^I_13/2_ (Er^3+^)	^3^F_4_ (Tm^3+^)
(a) 0.5 Tm	128	201	–	1440
(b) 0.5 Tm − 0.5 Er	103	165	1595	1620
(c) 0.5 Tm − 1.5 Er	53	109	646	1229
(d) 1.5 Tm	72	130	–	996
(e) 1.5 Tm − 0.5 Er	68	134	888	1260
(f) 1.5 Tm − 1.5 Er	62	128	775	1527

## Data Availability

The data presented in this study are available on request from the corresponding author.
